# Provision of cervical cancer services for women living with HIV, Uganda

**DOI:** 10.2471/BLT.23.290204

**Published:** 2024-03-12

**Authors:** Julius Namonyo Kalamya, Jennifer DeCuir, Sarah X Alger, Josephine Ninsiima, Joseph Kabanda, Patrick Komakech, Marvin Lubega, Grace Nantege, Estella Birabwa, Tamara Nsubuga Nyombi, Phoebe Namukanja, Steven Baveewo, Julius Ssendiwala, Jacqueline Calnan, Christina Mwangi, Mina Nakawuka, Gerald Mutungi, Lisa J Nelson, Emilio Dirlikov

**Affiliations:** a Division of Global HIV and TB, United States Centers for Disease Control and Prevention (CDC), United States Embassy Kampala, 1577 Ggaba Road, PO Box 7007, Kampala, Uganda.; b Epidemic Intelligence Service, CDC, Atlanta, Georgia, United States of America.; c Monitoring and Evaluation Technical Support, Kampala, Uganda.; d United States Agency for International Development, Kampala, Uganda.; e Clinton Health Access Initiative, Kampala, Uganda.; f United States Department of Defense, Kampala, Uganda.; g Ministry of Health, Kampala, Uganda.

## Abstract

**Objective:**

To describe the scale-up of cervical cancer screening and treatment for women living with human immunodeficiency virus (HIV), aged 25–49 years in Uganda, and to analyse the programme data.

**Methods:**

The health ministry targeted existing HIV clinics in a 2-year scale-up of cervical cancer screening services from October 2020. In preparation, we trained health workers to assess women attending HIV clinics for screening eligibility, provided either by human papillomavirus (HPV) testing and/or visual inspection with acetic acid. Clinic staff treated women with precancerous cervical lesions with thermocoagulation or referred women with suspected cancer to external services. We analysed data reported every 6 months for the number of clinics offering screening, screening uptake, the number of positive diagnoses and the number of women who received treatment.

**Findings:**

The number of HIV clinics offering cervical cancer screening services increased from 11, before the programme launch, to 1571. During the programme, screening uptake increased from 5.0% (6506/130 293) to 107.3% (151 872/141 527) of targets. The cumulative proportion of positive diagnoses was 5.9% (23 970/407 323) overall, but was much lower for screening offering visual inspection only compared with clinics offering HPV testing. Although the proportion of women receiving treatment if positive increased from 12.8% (53/413) to 84.3% (8087/9592), the World Health Organization target of 90% was not reached.

**Conclusion:**

We demonstrated marked increases, potentially replicable by other countries, in screening and treatment. These increases could be improved further by expanding HPV testing and same-day treatment of precancerous lesions.

## Résumé

**Objectif:**

Décrire l'intensification du dépistage et du traitement du cancer du col de l'utérus chez les femmes vivant avec le virus de l'immunodéficience humaine (VIH) et âgées de 25 à 49 ans en Ouganda, puis analyser les données du programme.

**Méthodes:**

Le Ministère de la Santé a ciblé les cliniques de prise en charge du VIH existantes dans le cadre d'un renforcement des services liés au cancer du col de l'utérus sur une période de deux ans, à partir d'octobre 2020. En amont, nous avons formé des professionnels de la santé à examiner les femmes fréquentant les cliniques du VIH afin de déterminer leur admissibilité au dépistage, par un test de détection du papillomavirus humain (HPV) et/ou une inspection visuelle après application d'acide acétique. Le personnel des cliniques a traité les femmes présentant des lésions précancéreuses du col de l'utérus par thermocoagulation, ou les ont renvoyées vers des services externes lorsqu'elles étaient susceptibles d'être atteintes d'un cancer. Nous avons analysé les données transmises tous les six mois concernant le nombre de cliniques proposant un dépistage, la participation au dépistage, le nombre de diagnostics positifs et le nombre de femmes ayant reçu un traitement.

**Résultats:**

Le nombre de cliniques du VIH disposant de services de dépistage du cancer du col de l'utérus a augmenté: 11 avant le lancement du programme, 1571 après. Au cours de ce programme, la participation au dépistage est passée de 5,0% (6506/130 293) à 107,3% (151 872/141 527) des cibles. Le pourcentage cumulé de diagnostics positifs s'élevait à 5,9% (23 970/407 323) au total; cependant, il était nettement moins important dans des cliniques effectuant uniquement des inspections visuelles que dans celles proposant un test de détection du HPV. Bien que le pourcentage de femmes ayant reçu un traitement en cas de diagnostic positif ait progressé de 12,8% (53/413) à 84,3% (8087/9592), l'objectif de l'Organisation mondiale de la Santé, fixé à 90%, n'a pas été atteint.

**Conclusion:**

Nous avons observé des hausses considérables, susceptibles de se reproduire dans d'autres pays, en matière de dépistage et de prise en charge. Ces hausses pourraient être accentuées par le développement des tests de détection du HPV et de traitement des lésions précancéreuses le jour même.

## Resumen

**Objetivo:**

Describir la ampliación del cribado y tratamiento del cáncer de cuello uterino para mujeres con el virus de la inmunodeficiencia humana (VIH) de entre 25 y 49 años en Uganda, y analizar los datos del programa.

**Métodos:**

El Ministerio de Salud se centró en las clínicas de VIH existentes en una ampliación de dos años de los servicios de cribado del cáncer de cuello uterino a partir de octubre de 2020. Como preparación, formamos a profesionales sanitarios para que evaluaran a las mujeres que acudían a las clínicas de VIH para determinar si cumplían los requisitos para someterse al cribado, mediante pruebas del virus del papiloma humano (VPH) o inspección visual con ácido acético. El personal clínico trató a las mujeres que presentaban lesiones cervicales precancerosas con termocoagulación o derivó a las mujeres con sospecha de cáncer a servicios externos. Se analizaron los datos comunicados cada seis meses sobre el número de clínicas que ofrecían el cribado, la aceptación del cribado, el número de diagnósticos positivos y el número de mujeres que recibieron tratamiento.

**Resultados:**

El número de clínicas de VIH que ofrecen servicios de cribado del cáncer de cuello uterino aumentó de 11, antes del lanzamiento del programa, a 1571. Durante el programa, la aceptación del cribado aumentó del 5,0% (6506/130 293) al 107,3% (151 872/141 527) de los objetivos. El porcentaje acumulado de diagnósticos positivos fue del 5,9% (23 970/407 323) en general, pero fue mucho menor en el caso de los diagnósticos en clínicas que solo ofrecían inspección visual en comparación con las clínicas que ofrecían pruebas del VPH. Aunque el porcentaje de mujeres que recibieron tratamiento en caso positivo aumentó del 12,8% (53/413) al 84,3% (8087/9592), no se alcanzó el objetivo del 90% que había fijado la Organización Mundial de la Salud.

**Conclusión:**

Se registraron aumentos notables, potencialmente reproducibles en otros países, en el cribado y el tratamiento. Estos aumentos podrían mejorarse aún más ampliando las pruebas del VPH y el tratamiento en el mismo día de las lesiones precancerosas.

## ملخص

**الغرض:**

وصف توسيع نطاق فحص سرطان عنق الرحم وعلاجه للسيدات اللاتي يعانين من فيروس نقص المناعة البشرية (HIV)، وتتراوح أعمارهن بين 25 و49 عامًا في أوغندا، وتحليل بيانات البرنامج.

**الطريقة:**

استهدفت وزارة الصحة العيادات الحالية التي تعالج فيروس نقص المناعة البشرية، وذلك في إطار توسيع نطاق خدمات فحص سرطان عنق الرحم لمدة عامين، اعتبارًا من أكتوبر/تشرين أول 2020. وفي سبيل الاستعداد لذلك، قمنا بتدريب العاملين في مجال الرعاية الصحية على تقييم السيدات اللاتي يترددن على عيادات فيروس نقص المناعة البشرية لتحديد مدى أهليتهن للفحص، والذي يتم تقديمه إما عن طريق فيروس الورم الحليمي البشري (HPV)، و/أو الفحص البصري باستخدام حمض الأسيتيك. قام فريق العمل في العيادات بمعالجة السيدات المصابات بالآفات التي تسبق حدوث سرطان عنق الرحم، عن طريق التخثير الحراري، أو إحالة النساء المشتبه في إصابتهن بالسرطان إلى الخدمات الخارجية. قمنا بتحليل البيانات المبلغ عنها كل 6 أشهر، وذلك لتحديد لعدد العيادات التي تقدم الفحص، ومعدل إجراء الفحص، وعدد التشخيصات الإيجابية، وعدد السيدات اللاتي تلقين العلاج.

**النتائج:**

ارتفع عدد عيادات فيروس نقص المناعة البشرية التي تقدم خدمات فحص سرطان عنق الرحم من 11 عيادة، قبل إطلاق البرنامج، إلى 1571 عيادة. وزاد معدل إجراء الفحص أثناء البرنامج من %5.0 (6506/130293) إلى %107.3 (151872/141527) من الأهداف. كانت النسبة التراكمية للتشخيصات الإيجابية %5.9 (23970/407323) بشكل عام، ولكنها كانت أقل بكثير بالنسبة للتشخيصات في العيادات التي تقدم الفحص البصري فقط مقارنة بالعيادات التي تقدم اختبار فيروس الورم الحليمي البشري. وعلى الرغم من أن نسبة السيدات اللاتي يتلقين العلاج، إذا كانت التشخيصات إيجابية، زادت من %12.8 (53/413) إلى %84.3 (8087/9592)، إلا أن هدف منظمة الصحة العالمية وهو %90، لم يتم الوصول إليه.

**الاستنتاج:**

لقد أظهرنا زيادات ملموسة، من المحتمل تكرارها بواسطة دول أخرى، في الفحص والعلاج. ويمكن تحسين هذه الزيادات بشكل أكبر من خلال توسيع نطاق اختبار فيروس الورم الحليمي البشري والعلاج في نفس اليوم للآفات التي تسبق الإصابة بالسرطان.

## 摘要

**目的:**

描述在乌干达为介于 25–49 岁之间的人类免疫缺陷病毒 (HIV) 女性患者扩大进行宫颈癌筛查和治疗的情况，并分析该计划的数据。

**方法:**

根据卫生部制定的目标，自 2020 年 10 月起，现有 HIV 诊所应在 2 年内扩大进行宫颈癌筛查的服务范围。为此，我们培训了一批卫生保健工作者，以评估前往 HIV 诊所接受筛查的女性，即采用人类乳头瘤病毒 (HPV) 检测方法和/或使用醋酸进行目视检查。诊所工作人员采用热凝固术治疗患有宫颈癌前病变的女性，或者将疑似患有癌症的女性转诊至外部服务机构。通过分析每 6 个月报告一次的数据，我们收集了提供筛查服务的诊所数量、筛查接受率、阳性确诊数量以及接受治疗的女性数量。

**结果:**

提供宫颈癌筛查服务的 HIV 诊所数量从 11 个（在开启该计划之前）增至了 1571 个。在该计划实施期间，筛查接受率目标值从 5.0% (6506/130,293) 增至了 107.3% (151,872/141,527)。所有阳性确诊病例累计占比 5.9% (23,970/407,323)，但是与提供 HPV 检测的诊所相比，仅提供目视检查的诊所的确诊率要低得多。虽然接受治疗的阳性确诊女性比例从 12.8% (53/413) 增至了 84.3% (8087/9592)，但是还未达到世界卫生组织设定的 90% 目标值。

**结论:**

研究表明，接受筛查和治疗的患者比例明显升高，其他国家可以效仿。通过扩大 HPV 检测范围和提高癌前病变患者当日治疗率，还可进一步提升上述比例。

## Резюме

**Цель:**

Описать расширение масштабов скринингового обследования и лечения рака шейки матки среди женщин, живущих с вирусом иммунодефицита человека (ВИЧ), в возрасте 25–49 лет в Уганде и проанализировать данные программы.

**Методы:**

Министерство здравоохранения нацелило действующие клиники по лечению ВИЧ на расширение услуг по скрининговому обследованию рака шейки матки в течение двух лет, начиная с октября 2020 года. В процессе подготовки этой статьи было проведено обучение медицинских работников по оценке женщин, посещающих клиники по лечению ВИЧ, на предмет соответствия скрининговому обследованию, которое проводится либо с помощью анализа на папилломавирус человека (ВПЧ), либо путем визуального осмотра с использованием уксусной кислоты. Сотрудники клиники лечили женщин с предраковыми поражениями шейки матки с помощью термокоагуляции или направляли женщин с подозрением на рак в другие службы. В рамках анализа данных, представляемых каждые 6 месяцев, было определено количество клиник, в которых проводится скрининговое обследование, количество положительных диагнозов и количество женщин, получивших лечение.

**Результаты:**

Количество клиник по лечению ВИЧ, предлагающих услуги по скрининговому обследованию рака шейки матки, увеличилось с 11 до 1571 до начала программы. За время действия программы охват скринингом увеличился с 5,0% (6506/130 293) до 107,3% (151 872/141 527) от целевых показателей. Суммарная доля положительных диагнозов составила 5,9% (23 970/407 323) в целом, но была значительно ниже для диагнозов, поставленных в клиниках, предлагающих только визуальный осмотр, по сравнению с клиниками, предлагающими тестирование на ВПЧ. Хотя доля женщин, получивших лечение в случае положительного результата, увеличилась с 12,8% (53/413) до 84,3% (8087/9592), целевой показатель Всемирной организации здравоохранения в 90% не достигнут.

**Вывод:**

По результатам скринингового обследования и лечения был отмечен значительный рост, который может быть воспроизведен в других странах. Эти показатели могут быть повышены за счет расширения тестирования на ВПЧ и лечения предраковых образований в тот же день.

## Introduction

Cervical cancer is a disease of the female reproductive tract caused primarily by oncogenic types of human papillomavirus (HPV). In 2020, it was the fourth most common cancer among women worldwide, with an estimated 604 000 new cases and 342 000 deaths.^1^ Low- and middle-income countries are disproportionately affected, with the highest levels of incidence and mortality occurring in sub-Saharan African countries.^1^ Women living with human immunodeficiency virus (HIV) are six times more likely to develop invasive cervical cancer compared with women without HIV infection.^2^ In 2021, the World Health Organization (WHO) released updated clinical guidelines for cervical cancer, recommending that women living with HIV aged 25–49 years are screened every 3–5 years.^3^

In Uganda, cervical cancer is the most common malignancy among women and the leading cause of cancer-related deaths.^4^ In 2010, the health ministry published a strategic plan for cervical cancer control, recommending annual screening for women living with HIV.^5^ However, a decade later less than 1% (11/1797) of HIV clinics supported by the United States President’s Emergency Plan for AIDS Relief (PEPFAR) providing antiretroviral therapy (ART) to women aged 25–49 years had introduced cervical cancer services. Among the HIV clinics that had introduced such services, uptake was low mainly because of a lack of resources for commodities and equipment due to competing health-care priorities. By 2018, more than 80% of about 6000 women referred each year for cervical cancer treatment to the Uganda Cancer Institute presented with either stage 3 or stage 4 cancer, of whom 70%–80% were HIV positive.^6^ In 2020, estimated HIV prevalence among women aged 15 years and older was 6.8%, and there were an estimated 810 000 women living with HIV.^7^ During that year, approximately 6959 Ugandan women were diagnosed with cervical cancer and 4607 women died of the disease.^4^

Given the high cervical cancer burden, low screening uptake and large population of women living with HIV, in October 2020 the health ministry launched an intensive scale-up of cervical cancer services among women living with HIV aged 25–49 years. In alignment with the WHO global strategy for cervical cancer elimination,^8^ the health ministry adopted a screen-and-treat approach to identify and treat precancerous cervical lesions in a timely manner. The key goals of this scale-up were to: improve patient literacy; build health workers’ capacity to provide high-quality cervical cancer services, especially the capacity of midwives and nurses to deliver screening and treatment of precancerous lesions; increase the availability and accessibility of high-quality cervical cancer services for women living with HIV; provide timely referrals to specialist care for suspected cervical cancer; and contribute to eliminating cervical cancer by 2030 through the 90:70:90 strategy (in which 90% of girls are fully vaccinated with HPV vaccine by age 15 years, 70% of women are screened with a high-performance test by age 35 years and again by age 45 years, and 90% of women identified with cervical disease receive treatment).^8^

We describe the nation-wide scale-up of cervical cancer screening and treatment in terms of its development and implementation. We also analyse the programme data for the 2-year period from its launch date, including the number of HIV clinics offering cervical cancer screening services; the number of women living with HIV screened; the number of women screened positive; and the number of women receiving treatment.

## Methods

### Development

Under the leadership of the Ministry of Health AIDS Control Program,^9^ the development of our scale-up of cervical cancer services for women living with HIV was supported by multiple internal and external stakeholders. We established a national coordination committee, bringing together experts from a wide range of relevant departments (e.g. noncommunicable diseases and reproductive health), local institutions (e.g. Uganda Cancer Institute and PEPFAR implementing partners) and international stakeholders (e.g. WHO Country Office in Uganda; PEPFAR; the Global Fund to Fight AIDS, Tuberculosis and Malaria; and the Clinton Health Access Initiative). We obtained the largest proportion of the required financial resources from PEPFAR, with additional support provided by the Global Fund and the Clinton Health Access Initiative. 

Our phased approach to the scale-up included: (i) materials preparation (March–August 2020), which included a review of cervical cancer strategy and guidelines, the development of tools and standard operating procedures, and the finalization of procurement plans, including commodity quantifications; (ii) training and delivery of commodities (September–December 2020), which included training required staff down to regional and district levels (starting with a national training-of-trainers), and HIV clinic preparations (including delivery of initial screening commodities and treatment equipment); (iii) HIV clinic-level activities (October 2020–March 2021), including training clinic staff and starting the screening and treatment services; and (iv) catching up from the effects of the coronavirus disease 2019 (COVID-19) pandemic (April–September 2021). During the final catch-up phase, some clinics were updated to improve the space available for screening and treatment, and implementation was fast-tracked, resulting in a large increase in access to screening and treatment.

### Implementation

We trained health workers to assess women living with HIV attending clinics for ART and other HIV services for screening eligibility at triage, and provide health education on cervical cancer. Screening is now offered as part of the routine service delivery, requiring informed consent from eligible women. Those who consent to be screened either (i) undergo visual inspection with acetic acid; or (ii) are tested for HPV followed by a visual inspection with acetic acid if positive. For the second method, a health-care provider instructs women on collecting a vaginal sample; samples are then tested using GeneXpert® (Cepheid, Sunnyvale, United States of America), following manufacturer instructions and health ministry guidelines.^10^ HIV clinics without GeneXpert® machines, or clinics with GeneXpert® machines but capacity limitations (e.g. lack of HPV cartridges, or competing testing needs with tuberculosis and COVID-19), offer screening by visual inspection with acetic acid only. Clinic staff schedule women who screen negative by either method for rescreening in 3 years.^3^

We also trained health workers to use the results of visual inspection with acetic acid to determine the appropriate treatment for women who screen positive. Those for whom eligible precancerous cervical lesions have been identified are treated with ablation (primarily thermocoagulation, with cryotherapy used at a few sites), ideally on the same day as screening. Women with lesions covering more than 75% of the cervix are ineligible for ablation, and are instead referred to regional hospitals for a loop electrosurgical excision procedure. Clinic staff schedule women who have undergone either type of treatment for rescreening after 1 year, to assess for lesion recurrence and potential treatment needs. Women with lesions potentially indicative of cervical cancer are referred to regional hospitals for biopsy and evaluation by gynaecologists. Hospital health workers refer women diagnosed with cervical cancer to the Uganda Cancer Institute or satellite sites for further management.

### Targets 

Of the 1797 PEPFAR-supported HIV clinics serving women aged 25–49 years, we targeted a total of 1789 (99.6%) for the provision or enhancement of cervical cancer services during years 1 and 2. The health ministry targeted 302 HIV clinics per 6-month period in year 1 (including the original 11), increasing to 592 then 593 for the two 6-month periods in year 2. To ensure that the most at-risk and age-eligible women benefitted from the scale-up, the health ministry set 6-month screening targets of 130 293 women living with HIV (annual total of 260 586 women) for year 1, and 141 527 women living with HIV (annual total of 283 054 women) for year 2. We estimated that the proportion screening positive for cervical cancer from HPV test and/or visual inspection with acetic acid would be 5%–25%.^11^ In alignment with the WHO global strategy for the elimination of cervical cancer,^8^ the health ministry programme aims to treat 90% or more of women diagnosed with cervical disease.

### Data analysis

We analysed data archived in the PEPFAR Monitoring, Evaluation, and Reporting Database for the 2-year scale-up. These data are routinely collected as part of HIV care and treatment services, and are reported every 6 months by PEPFAR-supported HIV clinics providing cervical cancer services. 

To quantify the scale-up of cervical cancer services at HIV clinics, we calculated the number of PEFPAR-supported HIV clinics serving eligible women living with HIV, determined as those that reported any data on cervical cancer screening during each 6-month period, as a proportion of the number of HIV clinics targeted for scale-up. 

We calculated the number of women living with HIV who were screened as a proportion of the target screening number for each 6-month period. We calculated the number of women living with HIV who screened positive by either HPV and/or visual inspection with acetic acid as a proportion of the total number of women living with HIV who were screened for each 6-month period, and compared this proportion with the expected proportion of women screening positive. Because screening method was not reported in the database, we assumed that: (i) women screening positive at HIV clinics without GeneXpert® machines received visual inspection only; and (ii) women screening positive at HIV clinics with GeneXpert® machines may have received HPV testing followed by visual inspection or, because of capacity limitations on HPV testing in some clinics, visual inspection only.

We calculated the number of women living with HIV who received treatment for precancerous cervical cancer lesions as a proportion of the number who screened positive for each 6-month period, and compared this proportion with the WHO treatment target of 90% or more.^8^

## Results

### Service provision

We observed that the number of HIV clinics serving eligible women living with HIV reporting cervical cancer screening increased from 11 before the health ministry scale-up to 1571 HIV clinics (87.8% of the cumulative 2-year scale-up target of 1789 HIV clinics) in September 2022 (Table 1). These 1571 HIV clinics providing cervical cancer services represent 87.4% (1571/1797) of all PEPFAR-supported HIV clinics serving eligible women. Of these 1571 HIV clinics, 5.3% (83) had GeneXpert® machines with which to provide HPV testing, and 94.7% (1488) provided screening via visual inspection with acetic acid only.

**Table 1:** Cervical cancer screening indicators among women living with HIV aged 25–49 years, Uganda, October 2020–September 2022

Indicator	No. achieved or diagnosed/target or sample size (%)				
	Oct 2020–Mar 2021	Apr 2021–Sep 2021	Oct 2021–Mar 2022	Apr 2022–Sep 2022	Cumulative results
Targeted clinics offering cervical cancer services	91/302 (30.1)	470/302 (155.6)	807/592 (136.3)	203/593 (34.2)	1 571/1 789 (87.8)
Screening uptake	6 506/130 293 (5.0)	102 912/130 293 (79.0)	146 033/141 527 (103.2)	151 872/141 527 (107.3)	407 323/543 640 (74.9)
Positive diagnoses	413/6 506 (6.3)	6 342/102 912 (6.2)	7 623/146 033 (5.2)	9 592/151 872 (6.3)	23 970/407 323 (5.9)
Positive diagnoses at clinics offering visual inspection with acetic acid only	60/3 266 (1.8)	2 495/74 688 (3.3)	4 279/109 433 (3.9)	3 993/110 958 (3.6)	10 827/298 345 (3.6)
Positive diagnoses at clinics offering HPV testing	353/3 240 (10.9)	3 847/28 224 (13.6)	3 344/36 600 (9.1)	5 599/40 914 (13.7)	13 143/108 978 (12.1)
Women receiving treatment if positive	53/413 (12.8)	3 581/6 342 (56.5)	5 695/7 623 (74.7)	8 087/9 592 (84.3)	17 416/23 970 (72.7)

HIV: human immunodeficiency virus; HPV: human papillomavirus.

### Screening

**Fig. 1:**
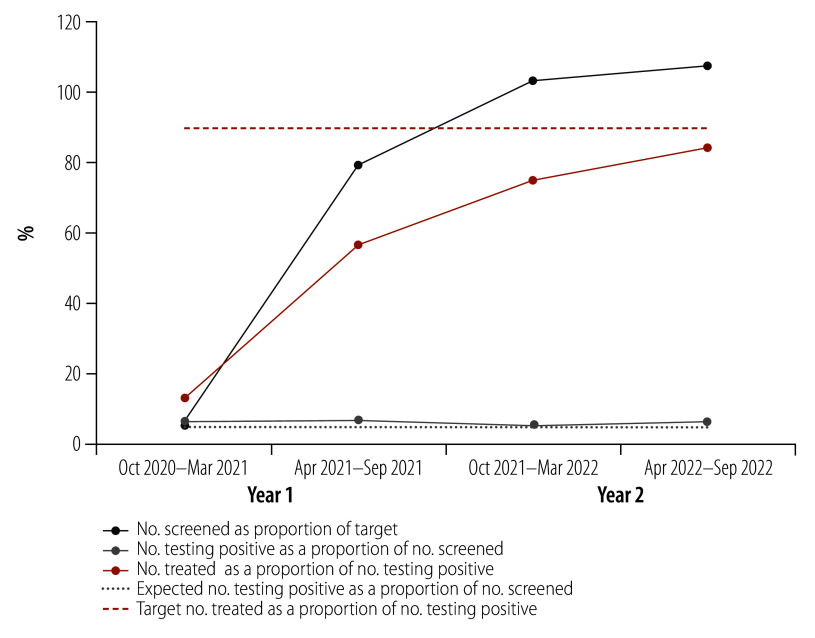
Cervical cancer screening indicators among women living with HIV aged 25–49 years, Uganda, October 2020–September 2022

Of the 1 215 789 people living with HIV receiving PEPFAR-supported HIV treatment on 30 September 2020, 44.8% (545 188) were women living with HIV aged 25–49 years, who were considered eligible for cervical cancer services. Only 5.0% (6 506/130 293) of the first 6-month screening target was achieved (Fig. 1; Table 1). However, screening uptake increased by the fourth 6-month period to 107.3% (151 872/141 527) of the target for that period. Overall, 407 323 women living with HIV were screened during the 2-year period, comprising 74.9% (407 323/543 640) of the cumulative target, and 74.7% (407 323/545 188) of eligible women living with HIV. 

HIV: human immunodeficiency virus.

### Positive diagnoses

During the 2-year period, 5.9% (23 970/407 323) of screened women living with HIV screened positive by either HPV testing and/or visual inspection with acetic acid; the number of positive diagnoses as a proportion of number screened was greater than 5% during each of the 6-month periods. HIV clinics without GeneXpert® machines, that is, clinics offering screening by visual inspection only, reported positive diagnoses of 3.6% (10 827/298 345) of women screened. However, HIV clinics with GeneXpert® machines, assumed to indicate screening by HPV testing followed by visual inspection or potentially visual inspection only, reported positive diagnoses of 12.1% (13 143/108 978) of women screened. 

### Treatment 

During the 2-year period, 72.7% (17 416/23 970) of women living with HIV who screened positive received treatment. The proportion of women receiving treatment increased over time from 12.8% (53/413) during October 2020–March 2021 to 84.3% (8087/9592) during April–September 2022 (Fig. 1; Table 1). 

## Discussion

Our scale-up allowed the successful integration of cervical cancer services into HIV clinics across Uganda, providing services to three quarters of all estimated eligible women living with HIV. We observed the number of women living with HIV who were screened and treated per 6-month period to increase by a factor of 23 (from 6506 to 151 872) and 153 (from 53 to 8087), respectively, during the 2-year period. The overall number of women diagnosed positive as a proportion of the number of women screened remained above the expected 5% threshold. 

Despite the successes of our programme, limitations and challenges remain. First, despite continual PEPFAR data quality assurance activities, data quality can be affected by reporting challenges (e.g. site-level electricity or internet outages preventing data transmission). Another potential bias in the data is that, given potential patient and provider treatment delays, it is quite possible that women could have been treated in a different 6-month period from that in which they screened positive, leading to artificially lower treatment rates.

Second, we observed a negative impact of the COVID-19 pandemic on service delivery. Because of reduced access to clinics by women and a disruption in the supply of commodities, only 5% of the first 6-month screening target was achieved. 

Third, the proportion of women screening positive at HIV clinics that only offer visual inspection with acetic acid is less than 5%. Visual inspection is subjective, that is, vulnerable to lower sensitivity depending on the experience of health-care providers^12^ with possible unrecognized precancerous lesions. To improve the quality of screening and limit the risk of false-negative results, experienced midwives have been providing support, supervision and additional training since October 2022. Expanded HPV testing capacity is also being planned.

Fourth, among women screening positive, the proportion of those receiving treatment remained below the WHO benchmark of 90%. This statistic could be improved by expanding the volume of same-day treatment of precancerous lesions. Same-day treatment was limited at the programme start by supply chain disruptions caused by the COVID-19 pandemic affecting the delivery of thermocoagulators. Despite COVID-19, all HIV clinics had the appropriate equipment by September 2021, and the proportion of women receiving treatment improved from this date. Another barrier to same-day treatment was reporting delays in HPV test results; such delays meant that women may have left the clinic before results become available, preventing same-day treatment and increasing loss to follow-up. To address these gaps, all women living with HIV who have screened positive according to either HPV testing and/or visual inspection with acetic acid are tracked to ensure they receive treatment.

To conclude, we have demonstrated the importance of planning and coordination with different stakeholders to strengthen the health-care system, including cervical cancer education for patients and capacity-building for health workers. The scale-up programme achieved marked increases in the number of women screened and, in those who screened positive, the number of women treated. These advances are expected to reduce morbidity and mortality due to cervical cancer among women living with HIV in Uganda. We anticipate that our experience of developing and implementing a national health-care programme aligned with WHO global benchmarks can be useful to other countries. 
